# Discrimination experiences of transgender individuals in healthcare: an interview study on the perspective of health professionals specializing in the treatment of transgender individuals

**DOI:** 10.1186/s12939-024-02313-1

**Published:** 2024-11-02

**Authors:** Tobias Skuban-Eiseler, Marcin Orzechowski, Florian Steger

**Affiliations:** 1 Institute of the History, Philosophy and Ethics of Medicine, Faculty of Medicine, Ulm University, Ulm, Germany; 2 kbo-Isar-Amper-Klinikum Region München, München-Haar, Germany

**Keywords:** transgender individuals, stigmatization, discrimination, healthcare, health professionals, transsexuality, ethics

## Abstract

**Background:**

Experiences of discrimination in healthcare lead to poorer mental and physical health for transgender individuals. There is evidence that trans-specialists, i.e. health professionals specializing in transgender care, are an important source of discrimination experienced by transgender individuals in healthcare. In this qualitative interview study, we explored the reasons for this possibly surprising finding by analyzing subjective views of trans-specialists on various issues related to discrimination of transgender individuals.

**Methods:**

We conducted 20 semi-structured, qualitative, exploratory interviews with healthcare professionals specializing in transgender care. Interview questions were developed based on an extensive literature analysis and results of previous research on the topic. The interviews were conducted online, were digitally recorded and transcribed. Data analysis was conducted using the methods of content analysis and thematic analysis.

**Results:**

The interviewees had great difficulty giving a consistent definition of the concept of gender identity. Most of them saw it as a self-determination of a transgender individual. Although herewith emphasizing the autonomy of transgender individuals, most trans-specialists felt that they had to be convinced of their patient’s transsexuality to carry out treatment. Most trans-specialists reported having had doubts about whether some transgender individuals were suffering from gender incongruence or not. There was also ambiguity among interviewees about whether transsexuality is a mental illness.

**Conclusions:**

We were able to identify specific topics that can cause discrimination experiences on the part of transgender individuals in their contact with trans-specialists. These include the vagueness of the construct of gender identity and the ambivalence between respect for the autonomy of transgender individuals and the validation of the diagnosis “transsexuality”. Also, uncertainties regarding the classification of transsexuality as a mental illness can lead to experiences of discrimination. Furthermore, our results imply that trans-specialists might remember own discriminatory behavior less than it actually took place. Our results can contribute to the development of specific measures to avoid discrimination experiences of transgender individuals in contact with trans-specialists. These should include a reflection on one’s own gender identity and training on mechanisms of discrimination.

## Background

Transgender individuals, i.e. those who do not, or to a lesser extent identify with the gender they were assigned at birth, continue to experience discrimination worldwide [1, 2]. Discrimination is influenced by social factors, such as a heteronormative environment, and may occur unconsciously [3]. Language also has a discriminatory effect. For example, the term ‘transsexuality’ is associated with pathologizing gender incongruence [4]. If we use this term in this article, this is solely due to the fact that ‘transsexuality’ is still used in Germany in the valid assessment guidelines of the health insurance companies and thus continues to be the valid term for the phenomenon of gender incongruence in German medical language. In this context, it is an encouraging development that the new versions of the psychiatric diagnosis systems ICD-11 [5] and DSM-5 [6] no longer use the term ‘transsexuality’. Furthermore, research shows that transgender individuals also experience discrimination in healthcare institutions [7–11].

From the perspective of transgender individuals, discrimination in healthcare can occur in different ways: by the use of pronouns that do not correspond to the gender with which a transgender individual identifies (misgendering), the use of the old first name of a transgender individual, which usually indicates the gender that was assigned at birth (deadnaming), disregard of a transgender individual’s special medical needs, by coercion to legitimize gender incongruence, harassment, restricted access to healthcare, disrespect of non-binary identification, open unfriendly behavior and the experience of being seen and treated only as an object of transsexuality and not as a person (objectivation) [12].

Experienced discrimination in healthcare, concerns about the ignorance of health professionals [13] or lack of consideration for transgender individuals in the heteronormative structures of the healthcare system [14], may lead to transgender individuals postpone seeking medical help or refuse medical care altogether [2, 15, 16]. This contributes to increased rates of physical [17] and psychological disorders [2, 18–20] among transgender individuals, particularly anxiety disorders [21, 22], social phobia [23], substance abuse and addictions [24], and eating disorders [25]. Moreover, transgender individuals have an increased risk of suicidal behavior [2, 26, 27].

Previously, we conducted an interview study on the topic of discrimination in healthcare from the perspective of transgender individuals. To our astonishment, the majority of our interviewees reported that the experiences of discrimination were made in contacts specifically with those health professionals that were specialized in the treatment of transgender individuals, referred to here as “trans-specialists” [12]. This term encompasses a range of disciplines involved in the medical transition process of transgender individuals: psychiatrists and psychotherapists, endocrinologists, surgeons, gynecologists, and speech therapists. Intuitively, one might assume that transgender individuals should not experience any discrimination in contact with trans-specialists since these professionals should have knowledge of the special needs of their patients and experience in contacts with transgender individuals. However, the results of our research show that this might not be the case.

There are numerous comparable studies on the topic providing the viewpoint of transgender individuals. However, to our knowledge, there is no study that focuses on the perspective of trans-specialists and their outlook on experiences of discrimination of transgender individuals in healthcare. Therefore, the goal of the research presented in this paper was to gather and analyze subjective views of trans-specialists on the topic of discrimination of transgender individuals in healthcare. As a result, we aimed to identify specific measures to prevent transgender individuals from experiencing discrimination when in contact with trans-specialists in the future.

Since it is the very nature of gender incongruence that a transgender individual’s gender identity does not correspond to the one assigned at birth [28], and since addressing and discussing gender identity is an important element of gender-affirming care [29], we assume that the understanding of gender identity is an essential element in the interaction between transgender individuals and trans-specialists. We were interested in (I) what trans-specialists understand by gender identity and (II) whether they assume that they need to be convinced of a transgender individual’s transsexuality in order support a transition. As people suffering from mental illnesses are severely discriminated against [30], it can be assumed that the identification of transsexuality as a mental illness may lead to experiences of discrimination on the part of transgender individuals. For this reason, we wanted to know (III) whether trans-specialists do understand transsexuality as an illness or not. Finally, we wanted to find out (IV) to what extent trans-specialists themselves report having discriminated against transgender individuals, (V) what reasons they see for experiences of discrimination against transgender individuals in contacts with trans-specialists and (VI) what measures they propose to avoid experiences of discrimination against transgender individuals in contacts with trans-specialists.

## Methods

In order to answer these questions, we conducted a series of semi-structured, qualitative, exploratory interviews with healthcare professionals specializing in transgender care. This method of research is especially suitable for capturing the subjective views of interview partners on the topic under study based on their background and individual experiences. Moreover, exploratory interviews provide certain flexibility in the conduct of the interviews; they allow asking ad hoc question, give possibility to clarify statements or focus on specific issues mentioned by the interlocutors [31].

The investigation was designed by an interdisciplinary team of researchers comprising of a board-certified psychiatrist (T.S-E.), ethicist and political scientist (M.O), and physician and expert in ethics and history of medicine (F.S.). The interview questions were developed on the basis of an extensive literature analysis and results of previous research on the topic. Limitations of the chosen method, as well as the issues of validity and bias of the results were discussed within the research team prior to conduct of the investigation.

In order to access possible interview partners, a purposive sampling design was used [32]. Contact with interlocutors was established through quality circles associating medical professionals with trans-gender specialization. The researchers contacted possible interview partners directly via email. The participants were informed about the research’s purpose, method, and persons responsible for conducting the investigation. Moreover, the participants were informed about voluntariness of participation, confidentiality of the data collected and possibility of withdrawal at any time up to the moment of anonymization of their interviews. Interview dates were then scheduled with individual participants. The interviewees took part in the research on voluntary basis and were not compensated in any way for their participation.

The interviews were conducted online, through a secured digital communication platform in the native language of the participants (German). All interviews were conducted by one researcher, who had knowledge on the research topic, qualitative research methods, and experience in organization and conduct of scientific interviews. The basis of the interviews was a set of 20 questions, which were complemented by ad hoc questions for clarification of the respondents’ statements.

The interviews were digitally recorded and transcribed by an external transcription service, which has an extensive experience in handling sensitive data in the field of health research. After transcription, the written transcripts were anonymized and recordings were deleted. The transcripts served as a basis for further analysis. Data analysis was conducted using the methods of content analysis [33] and thematic analysis [34, 35]. For this purpose, the responses were reduced to core elements and statements. Particular topics were formulated deductively and inductively on the basis of the questionnaire’s topics and the content of the interviews. These thematic categories were formulated based on the prevalence of the themes in the interviews’ content. Individual statements were manually coded, extracted, and systemized through clustering into main topics and subtopics. Quotes representative for particular topics were translated into English by the authors. The results in each category are presented in a narrative manner.

## Results

### Demographics

We conducted interviews with a total of 20 trans-specialists. We were able to recruit these via two large networks of practitioners, one of which is based in Munich and the other in Berlin. 16 interviewees came from the Munich network and four from the Berlin network. The majority of 19 interviewees worked in private practices, the interviewed surgeon worked in a hospital. The interviewees were not direct colleagues. However, as the two networks are designed to improve care pathways for trans individuals, some of the interviewees had professional contacts with each other from time to time. As there is no binding and officially recognized further training for the care of trans individuals in Germany, the status of ‘trans-specialist’ of our interviewees resulted from the clinical focus on the care for trans individuals. We interviewed three child and adolescent psychotherapists, four adult psychotherapists, three psychotherapists who treat children, adolescents and adults, three psychiatrists, five doctors specializing in endocrinology/internal medicine/general medicine, one gynecologist and one surgeon. The interviewees had been working in their respective professions for an average of 17.8 years (8 to 49 years) and had been treating transgender individuals for an average of 10.2 years (4 to 26 years). Of our interviewees, 13 declared themselves as females and 7 as males.

### Attitudes towards gender identity

We asked our interviewees what they understood by gender identity. Most of them were initially confused by this question and found it difficult to answer. More than half of the interviewees thought that gender identity was a vague feeling that caused transgender individuals to have doubts about their biological gender. Interviewee 1 described this as follows:

Gender is *“[…] the inner feeling of belonging to one’s own sex*,* i.e. to one’s biological sex. So*,* well*,* yes*,* the inner feeling of belonging to a gender*,* whether that is my biological gender or not or whatever. So*,* it’s a feeling*,* it’s actually a feeling for me; I don’t usually read many studies. ‘I’m not a book’*,* I always tell my patients. I don’t always know any figures either. I’m such a gut person.”*

Some interviewees understood gender identity to be a form of certainty that somehow manifests in transgender individuals. Some interviewees had the impression that the construct of gender identity was too complex to define it. Some interviewees stated that gender identity is primarily the result of social factors. Interviewee 3 expressed this as follows:

*"I believe that even this personal categorization, am I a man, am I a woman, is very much dependent on society. […] So you can’t detach it from the social trend […] They don’t live in a vacuum, they see what is expected of boys, what is expected of girls. And I think they then reinforce this and try to validate themselves, so to speak, in their gender perception."*

Some interviewees understood gender identity to be primarily the result of biological factors. In this context, particular reference was made to the genetic determination of gender identity. Interviewee 8 mentioned that a biological definition of gender identity might be sanctioned in the field of trans-specialists:

*"Gender identity is first and foremost what you are biologically. For me, gender is biologically determined by genetics and genitalia. However, I know that not all people see it that way. They also see gender as a social gender. In other words, how you were brought up, how you were molded by society, how you classify yourself and so on. And I respect that too. That’s an individual point of view, so to speak, and I accept it, but it’s not my own opinion. […] So of course, if I were to say that openly somewhere, I would immediately get a headwind, even from the patients, yes. […] So your gender is actually what you say in chromosomes or what you have on your genitals, yes? That’s what it’s based on. Because that’s only relevant for reproduction and nothing else*.*"*

Some interviewees thought that gender identity was the result of experiencing a physical discrepancy. Trans identity would arise when physical gender attributes were perceived as inappropriate. One person defined gender identity as the result of a process of self-determination. One interviewee expressed doubts as to whether a construct called “gender identity” exists at all.

We also asked the interviewees whether they understood gender identity as a binary phenomenon, in that two genders are assumed (male and female), or whether they had other ideas about the internal structure of gender identity. Most interviewees stated that there were other gender identities besides a male and female identity. Some interviewees admitted they only assumed the existence of the gender identities of man and woman.

We were interested in our interviewees’ assessments of how the self-identification process works for transgender individuals. We asked, how transgender individuals would recognize that their perceived gender identity does not correspond to that which appears to be predetermined by their physical gender characteristics. More than half of the interviewees said that this process of self-recognition was the result of a diffuse inner feeling of foreignness. Interviewee 5 expressed this as follows:

*“So*,* I think that the process of self-recognition is triggered by feelings of being uncomfortable*,* unwell*,* out of place*,* out of tune*,* yes? That this triggers this process*,* yes? That I have the feeling that this body*,* this role somehow makes me feel uncomfortable.”*

Some interviewees believed that trans identity arises from experiences of physical discrepancy. Several interviewees saw experiences of social discrepancy as important. Interviewee 18 formulated it as follows:

*"I think it is more the perception of how you might want to appear to others. How you see yourself in the social structure. That you would perhaps prefer to take on the other role*.*"*

One interviewee described the process of self-recognition being a process of self-examination and reflection. This process would lead to a certainty on the part of transgender individuals not to have the gender identity that seemed to be predetermined by their physical characteristics. Some interviewees formulated that gender identity could also be constituted by consuming information available in the media. Interviewee 11 said:

*"Nowadays you can probably just type certain feelings into the internet and then you can get a lead or a trace of what’s going on with you […]."*

One interviewee (interviewee 13) was convinced that there could never be certainty regarding one’s own gender identity. Hence, trans identity could never reliably be recognized:

*"You never have certainty and I also believe there is no such certainty. These are snapshots. They are snapshots of life phases."*

We wanted to know from our interviewees whether they thought that trans-specialists had to be convinced of a transgender individual’s trans identity to carry out appropriate treatment. The majority of interviewees believed a trans-specialist’s conviction regarding a transgender individual’s trans identity was a prerequisite for treatment:

*“In my counter-transference*,* I also need the certainty that I can go along with it. And if I don’t feel that*,* then it just doesn’t work for me.”* (interviewee 3).

*“And then I really have to stand behind it. I think I have to be able to understand it. I don’t have to be enthusiastic about it*,* I don’t know. But I have to be able to understand it in broad strokes […].”* (interviewee 10).

*“I have to believe it. So*,* I think we definitely have to believe it.”* (interviewee 13).

Some interviewees believed that trans-specialists do not have to be convinced of the trans identity of their patients to carry out proper treatment. One interviewee (interviewee 7) attributed this to the fundamental impossibility of being able to judge a person’s trans identity at all:

*"Well, at least I can’t prove it from the outside or use any diagnostic tools to make it clear that this person is trans."*

Finally, we wanted to know whether the interviewees had ever experienced doubts about a transgender individual’s trans identity. Nearly all interviewees reported having had such doubts. Most interviewees had experienced such doubts at least several times or even often, several interviewees only in a few cases. Various aspects were mentioned as reasons why such doubts might arise. Some interviewees attributed doubts to the simultaneous presence of other serious psychiatric illnesses, other saw uncertainty or ambiguity on the part of patients as a major source of doubt, a few named specific groups regarding to which doubts on trans identity might occur. Older people were mentioned, but also younger people, because it could not be ruled out that their identification with a certain gender identity might still change; people with migration background were also mentioned, because uncertainty could arise as to whether the diagnosis of transsexuality could only be sought because they want to secure their stay in the country. One interviewee cited clichéd ideas about binary gender on the part of transgender individuals as a major source of doubt (interviewee 8):

*"Yes, why can’t a boy like unicorns? Or the color pink? Why can’t a girl like climbing trees? Well, first of all, there’s the cliché in your head. And then: ‘Do I fit the stereotype? Oh no, then there must be something wrong with me’. Instead of saying: ‘What kind of society do we live in that is so divided? A boy can also like unicorns. Why not?"*

One interviewee expressed relief for not belonging to a professional group that must diagnose transsexuality. One interviewee stated that doubts about any person’s gender identity were not appropriate, as nobody could judge on what gender identity a certain person does identify with.

### Transsexuality: an illness?

We wanted to know from our interviewees whether they categorize transsexuality as an illness. The overwhelming majority of interviewees were certain that transsexuality should not be categorized as an illness. Several considered transsexuality to be a variant of nature, some believed it to be an indeterminate difference whatsoever, and one interviewee described the categorization as transsexual as an outdated classification anyway. Nearly half of the interviewees conceded that the classification as an illness was important, as otherwise certain medical interventions would not be financed by the health insurance companies. Several interviewees related the phenomenon of transsexuality to other pathological conditions. One interviewee saw gender dysphoria as illness, not transsexuality per se. Some interviewees emphasized that transsexuality itself leads to suffering, as transgender individuals experience being very different compared to most other people. One interviewee (interviewee 8) had the impression that transsexuality might be the symptom of another underlying illness:

*"According to the depth psychology schema, I would say […] identity diffusion. Traumatization and personality disorder are usually involved. I hardly know anyone, maybe one or two, who wouldn’t fulfil the criteria of a personality disorder or traumatization."*

### Experiences of discrimination by transgender individuals

Almost all interviewees stated that they regularly discussed discrimination experienced by transgender individuals. We asked our interviewees whether they thought they themselves had ever discriminated against transgender individuals in the course of their clinical work. The overwhelming majority of interviewees stated that they could remember having possibly discriminated against transgender individuals. However, all interviewees emphasized that such incidents had occurred only rarely and unintentionally at best and that they assumed that any experiences of such discrimination on the part of transgender individuals were of a minor nature at best. The following aspects were named as the cause of possible experiences of discrimination: personal disappointment by the therapist or overly critical enquiries about trans identity, misgendering, refusal to attest a transsexuality, too much binary thinking on the part of trans-specialists, too less attention to the concerns of transgender individuals. One interviewee just stated:

*“Well*,* I think that’s part of being human*,* that sometimes you probably discriminate against someone by mistake.”* (interviewee 15).

We wanted to know from our interviewees what grounds they suspected are the reasons for transgender individuals’ experienced discrimination in therapeutic contacts with trans-specialists. We identified nine different reasons suggested by our interviewees:

1. Trans-specialists would sometimes have to take on the role of gatekeepers, even if they did not want to. They decide whether medical interventions during transition can take place.
2. Trans-specialists might have chosen their profession only because they themselves reject transsexuality.

*“Then perhaps it’s transference and counter-transference that goes on non-verbally*,* the doctor transmits without realizing it. Yes. There are people who deal with trans right now because it’s what they dislike the most […].“* (interviewee 4).

*„Indeed*,* I think there are a lot of older people who do it. And they’ve also experienced other phases*,* so to speak*,* and are still involved. Then I would think*,* but maybe that’s really not differentiated enough*,* that there is often something inherently homophobic in analytical therapy.“* (interviewee 7).

(3) Experiences of discrimination by transgender individuals could also be caused by a strong power imbalance between trans-specialists and transgender individuals or by excessive hubris on the part of trans-specialists. As transgender individuals are particularly vulnerable, the power imbalance with trans-specialists is particularly considerable. The interviewees presumed that this could lead to transgender individuals being used by trans-specialists to increase their own self-confidence.
(4) One interviewee surmised that one reason for transgender individuals experiencing discrimination could be that the psychotherapeutic care of transgender individuals was easy to do. As a result, there might be a possibly unjustified feeling of being particularly competent on the part of trans-specialists leading to experiences of discrimination by transgender individuals.
(5) A certain rigidity on the part of trans-specialists could be experienced as discriminatory. An inflexible orientation towards questioning habits and an unjustified request for intimate details could contribute to experiences of discrimination for transgender individuals. Moreover, the fact that many transgender individuals do not voluntarily enter contact with trans-specialists, especially psychotherapists and psychiatrists, but because they need certain statements and confirmations in order to be able to carry out the transition process could also contribute to experiences of discrimination:

*“And when a specialist asks why*,* why*,* why*,* why*,* then I think this wound is torn open*,* yes? That ultimately*,* I can’t explain it*,* it’s just my experience*,* yes? And that’s why it could be that they feel offended or hurt.”* (interviewee 5*)*.

*“’I’m supposed to tell him something about myself. I’m not there voluntarily*,*’ […] of course it’s often a difficult conversation situation*,* yes? That may be."* (interviewee 10).

(6) Insecurity or lack of knowledge on the part of trans-specialists was also mentioned by our interviewees as a reason why transgender individuals can experience discrimination in therapeutic contacts with trans-specialists. A behavior that appears arrogant could be a sign of insecurity.
(7) One interviewee (interviewee 5) cited the vagueness of the construct of gender identity as a reason for transgender individuals’ experiences of discrimination. It would be almost impossible for them to explain, why they feel they are trans. In such context, unsatisfactory conversational situations could arise, in which transgender individuals struggle for an understanding that just cannot be generated.
(8) Binary thinking on the part of trans-specialists could be experienced as discriminatory.
(9) One interviewee (interviewee 13) cited non-binarity as a particular challenge. In this context, there could be wishes on the part of transgender individuals (such as microdosing during gender affirming hormone therapy) that are difficult to fulfil from a medical perspective. If such wishes were then rejected, experiences of discrimination on the part of transgender individuals could arise.

Finally, we asked the interviewees what should be done to prevent transgender individuals from experiencing discrimination in therapeutic contacts with trans-specialists. The following options were mentioned: (1) mandatory curricula and certifications to substantiate designation as ‘trans-specialist’; (2) mandatory self-experience sessions on own gender identity for trans-specialists; (3) education on interactional processes leading to experiences of discrimination; (4) better adherence to medical guidelines in order to establish reliable therapeutic processes; (5) regular intervision; (6) offering regular advanced training for trans-specialists; (7) more education regarding trans identity for the society as a whole; (8) structural measures such as the establishment of gender officers in clinics, provision of non-binary toilets, noting and respecting transgender individuals’ preferred pronouns; (9) discussion of wishes and expectations as well as the different roles at the beginning of a treatment; (10) strengthening contacts between trans-specialists and the trans community.

## Discussion

One of the main aims of this study was to find out to what extent trans-specialists contribute to transgender individuals’ experiences of discrimination and what special features characterizing the therapeutic contacts between transgender individuals and trans-specialists can increase perceived discrimination on the part of transgender individuals.

### Demographics

We conducted interviews with a total of 20 trans-specialists. The majority of the interviewees declared themselves as females (13 interviewees) in comparison to interviewees who declared themselves as males (7 interviewees). This reflects the fact that the proportion of persons who identify as female in medical disciplines and in psychotherapy has been steadily increasing for years. Currently, there are now more doctors and psychologists working in Germany that identify as female than colleagues that identify as male [36]. It must be mentioned here that the corresponding statistics are still organized in binary form, so that non-binary health professionals cannot be recorded. We succeeded in interviewing almost all specialist disciplines involved in trans care. The overrepresentation of psychologists and psychiatrists is due to their importance in the transition process of transgender individuals, at least in Germany. With an average length of employment of 17.8 years and over 10 years of focusing on the treatment of transgender individuals, our interviewees can be regarded as very experienced.

### Attitudes towards gender identity

Since transsexuality is to be understood as a phenomenon which particularly affects gender identity [28], we were interested to find out what trans-specialists understand by gender identity and to what extent a consistent concept of this phenomenon can be found among trans-specialists. However, we were unable to identify such consistent concept. Rather, it became clear that most interviewees had difficulties formulating what they understood by “gender identity”.

The variety of ideas about gender identity and its identification was also reflected in the ideas of our interviewees as to whether gender identity is a binary phenomenon.

When the term “gender identity” first appeared in scientific discourse, it was characterized by binary thinking. It was coined by Robert Stoller and Ralph Greenson in the year 1963 [37]: „Gender identity is the sense of knowing to which sex one belongs, that is, the awareness ‘I am a male’ or ‘I am a female’“ [38], „Gender identity refers to one’s sense of being a member of a particular sex; it is expressed clinically in the awareness of being a man or a male in distinction to being a woman or a female“ [39]. These definitions lost much of their precision when gender identities were observed beyond the binary of male and female and when people began to no longer identify on a gender spectrum at all. Hence, now gender identity is an ill-defined term [40]. Rather, it is understood as a concept beyond biological attributes [41], being the result of a self-attribution process [42]. With the increasing social awareness of gender identity, self-attributions are becoming increasingly relevant. However, as self-attributions arise from highly individual and subjective experiences, the self-attributed gender identities prove to be very disparate in many ways, can be traced back to numerous different categories, often differ from biomedical definitions and thus may run counter to one-dimensional notions of gender identity [43]. Gender identity is understood as a complex construct with many different facets, which is not limited to binary concepts of man/woman or male/female [44, 45]. The fact that our interviewees were unable to provide clear definitions of gender identity can be explained on the one hand by the complexity of the construct, but also by scientific uncertainties that exist about gender identity. Too little is still known about the nature of gender identity and the factors that determine it. Even though biological factors are frequently cited as the main sources of gender identity, scientific data on these biological factors is considered not to be convincing [46]. However, there are also competing scientific assessments that state that biological factors are of paramount importance in determining a person’s gender identity [47]. This uncertainty was also evident in our interviews, in which some of our interviewees believed biological factors were decisive in the genesis of gender identity, with the remaining interviewees considering other factors to be more important.

Our interviews revealed very different ideas regarding the self-identification process of transgender individuals. These ranged from the view that it is not possible to speak of a realization of one’s own gender identity, to the idea of diffuse self-identification processes or observations of physical or social discrepancies.

This article is less about discussing what is meant by gender identity or how to recognize it. However, we show that the ideas about gender identity and the corresponding self-recognition processes on the part of trans-specialists are highly disparate. This may contribute to situations in which transgender individuals are more likely to experience discrimination. There are various reasons for this. On the one hand, it has already been shown that ignorance regarding transsexuality and trans care is a source of discrimination [48–50]. If there is too little knowledge about a particular issue, the trans-specialist’s own ideological assumptions or cultural stereotypes might control their behavior towards transgender individuals [51, 52]. Gender identity, the phenomenon that is certainly to be regarded as central in the context of the care of transgender individuals, apparently proves to be poorly defined and one about which there is no consensus, at least among the trans-specialists we interviewed, either with regard to the definition or the associated process of self-recognition. Given the current state of research, it is questionable whether we even have enough information to define gender identity clearly and adequately. This lack of clarity could also be formative in therapeutic contacts with transgender individuals and promote experiences of discrimination according to the mechanisms mentioned above. Implicit ideas of gender identity could be more powerful in therapeutic contacts than desired. This is because our attitude towards other persons is very much determined by implicit mechanisms. Attitudes, judgements, prejudices and stereotypes do not necessarily have to be conscious and explicit in order to control a person’s behavior. In many cases the cognitions resulting in a certain behavior remain unconscious [53]. This may lead to a different behavior towards certain groups of people [54, 55]. Greater clarity on the different aspects of gender incongruence can significantly improve the experiences of transgender individuals in healthcare [14, 56]. Since gender identity is obviously not clearly defined, the greatest sensitivity should be shown by trans-specialists in discussions on gender identity. This also means that strict care should be taken not to control the contact with transgender individuals through one’s own implicit ideas. When asking about gender identity, one should avoid defining or implicitly assuming certain categories in advance. Therefore, it has been suggested that when asking about gender identity, as few guidelines as possible should be given and the interviewees should literally be placed in front of a blank sheet of paper on which they should formulate their answer [57]. In addition to such interventions, however, it seems to be crucial for trans-specialists to reflect on their own implicit ideas, which, if they become aware of them and are willing to modify them, can also prove to be changeable [58, 59].

Most of our interviewees stated that the conviction that their patients are actually transsexual is a prerequisite for treatment. Only a minority of interviewees did not share this opinion. At the same time, most interviewees stated that they had already experienced doubts about their patients’ transsexuality. For our interviewees, these doubts were based on (1) the simultaneous presence of other serious psychiatric illnesses, (2) patients belonging to certain groups of people such as older or younger people or people with migration background. Regardless of the reasoning, it is remarkable that most of our interviewees assumed that a transsexuality can be recognized essentially or exclusively by the person concerned, but at the same time expressed occasional doubts about the reality of self-attributed transsexuality. If one were to take it seriously that only the person concerned can recognize whether he or she is experiencing gender incongruence, then every self-attribution of transsexuality by transgender individuals should actually be taken sincerely and accepted. Doubts about the existence of transsexuality would then consequently not be appropriate at all and it would also be irrelevant whether a trans-specialist believes a transgender individual to be transsexual or not. A striking discrepancy is noticeable: on the one hand, gender incongruence is seen as an autonomous self-attribution of transgender individuals. On the other hand, there seems to be a certain claim on the part of trans-specialists to be convinced of a transgender individual’s transsexuality and to dispel any doubts in this regard. It remains to be discussed whether this claim by trans-specialists is justified. However, this can lead to situations in which experiences of discrimination may become more likely for transgender individuals. As part of their identification process, transgender individuals may experience phases in which they are irritated by their gender incongruence. When they embark on the path of transition, they have usually left these irritations behind them [60]. Doubts on the part of trans-specialists could then reactivate identification steps that have already been mastered and make transgender individuals feel that their development that has already taken place is not being taken seriously enough. In addition, doubts on the part of trans-specialists could also be misinterpreted as gender stereotypical attitudes, which are not uncommon among health professionals and can considerably impair the quality of therapeutic contacts [61]. Non-acceptance of transsexuality is a phenomenon that transgender individuals often encounter. Hence, transgender individuals make less use of healthcare [62], and suffer from poorer physical, mental and sexual health [63, 64]. Every effort should be made not to give transgender individuals the impression that their transsexuality is not accepted when they are in contact with trans-specialists. In our opinion, this would require a transparent approach to any doubts in the therapeutic process. Moreover, trans-specialists should urgently reflect on the extent to which any doubts about the existence of transsexuality can be justified at all.

### Transsexuality: an illness?

Regarding the question of whether transsexuality should be categorized as an illness, the majority of our interviewees were of the opinion that this is not the case. Nevertheless, a certain discrepancy emerged: a significant number of our interviewees was nevertheless convinced that the classification of transsexuality as an illness was of enormous importance in the context of a health insurance-funded healthcare system. Hence, trans-specialists seem to be caught between two extremes: on the one hand, the prevailing opinion is that transsexuality is not a disease, but on the other hand, at least in Germany, a disease must be diagnosed in order for health insurance companies to cover the costs. Categorization of transsexuality as an illness creates a precarious legal status for transgender individuals. It has also led to numerous human rights violations, although there is sufficient evidence that transsexuality is not a disease [65]. If there is a lack of clarity in the therapeutic contact between a transgender individual and the trans-specialist as to whether transsexuality is or should be an illness, we believe that this can lead to experiences of discrimination of transgender individuals. It would be significantly less discriminatory if transgender individuals could make use of medical interventions without having to rely on a diagnosis of transsexuality [66]. The fact that the diagnosis “gender identity disorder” has now been replaced by the diagnosis “gender dysphoria” in DSM-5 [6] and that the coding “gender incongruence” has been removed from the chapter “Mental and Behavioral Disorders” in ICD-11 [5], may give some hope. Nevertheless, there is still a great need worldwide to de-pathologize transsexuality [67], not least in order to prevent experiences of discrimination on the part of transgender individuals.

### Experiences of discrimination by transgender individuals

Almost all of our interviewees stated that experiences of discrimination of transgender individuals were regularly discussed with their patients. Most of the interviewees admitted that they might have unintentionally discriminated against transgender individuals. However, they were all convinced that such incidents were the exception rather than the rule and that any experiences of discrimination by transgender individuals have been minimal at best. This is in clear contrast to the results of our previously mentioned study, in which a large proportion of the transgender individuals interviewed stated that they had experienced discrimination from trans-specialists in particular [12]. We do not want to claim that the statements of the trans-specialists we interviewed regarding their own discrimination are to be doubted. Nevertheless, there are certainly indications in social science that our social behavior is to a large extent not under our conscious control, but is controlled by implicit and unconscious processes [68]. These can be influenced by stereotypes, which have a longer history and therefore a higher frequency of activation than newly acquired personal beliefs. Therefore, not only an intentional inhibition of automatically activated stereotypes is necessary, but also an activation of new personal beliefs if one wants to show a reaction that is not characterized by prejudices or stereotypes [69]. Interestingly, people are often not even aware of the extent to which the behavior they display might be driven by implicit stereotypes [70]. Hence, it cannot be ruled out that trans-specialists are a more frequent source of discrimination experienced by transgender individuals than our interviewees suggested. We believe that there is a great need for trans-specialists to deal explicitly with mechanisms of discrimination and implicit stereotypes to minimize or completely avoid transgender individuals’ experiences of discrimination.

We asked our interviewees what they thought were the reasons why trans-specialists could be a source of discrimination for transgender individuals. These largely corresponded to the reasons that transgender individuals had given us previously [12]. There was agreement on the following factors: (1) possible rejection of transsexuality by trans-specialists, (2) asymmetrical distribution of power in therapeutic contacts between trans-specialists and transgender individuals, (3) ignorance and (4) binary thinking on the part of trans-specialists. In addition, our interviewees mentioned the following possible reasons: (1) trans-specialists acting as gatekeepers, (2) unjustified feeling of competence by trans-specialists, (3) inflexibility and rigidity of trans-specialists, (4) vagueness of the construct of gender identity and (5) inability or ignorance in dealing with non-binarity. In our previous study, transgender individuals also mentioned: (1) structural deficits, (2) current political debate, (3) the feeling that transgender individuals could be a burden on society, (4) objectification of transgender individuals, (5) misogyny and androcentrism and (6) discrimination against transgender individuals in order to avoid being the subject of discrimination oneself [12]. To our knowledge, this is the most comprehensive compilation of possible reasons why transgender individuals may experience discrimination in contacts with health professionals, and in particular with trans-specialists. Trans-specialists should urgently address these factors as part of structured training, intervision or supervision as regularly as possible. This might counteract trans-specialists becoming the source of discrimination experienced by transgender individuals.

Finally, we asked our interviewees for their opinion on what interventions they thought were necessary to prevent transgender individuals from experiencing discrimination by trans-specialists. There are already numerous suggestions in the scientific literature as to how experiences of discrimination against transgender individuals in healthcare can be prevented or minimized. These include the demand for specific training on mechanisms of discrimination [71, 72], increased research on discrimination against transgender individuals in healthcare [73], political and institutional changes [74], the generation of respect for transgender individuals and their special needs [75], the creation of a trans-inclusive environment [74] and the strengthening of co-operation with the trans community [76]. In addition to these measures, the trans-specialists we interviewed also mentioned mandatory seminars in self-awareness regarding own gender identity, better adherence to medical guidelines, regular intervision and the discussion of wishes and expectations of the various roles at the beginning of treatment. The measures mentioned by the interviewees seem to address different reasons that, according to our interlocutors, are responsible for transgender individuals’ experiences of discrimination. Self-experience sessions, education on processes of discrimination and regular intervision could address all the reasons mentioned. The introduction of mandatory curricula, regular advanced training and more education regarding trans identity could demonstrate the care for transgender individuals in all its complexity and thus also eliminate uncertainties and knowledge deficits. Better adherence to guidelines could improve the rigidity of some trans-specialists. Structural measures could help trans-specialists to question overly binary thinking. A discussion of wishes and roles prior to counselling or therapy could help to avoid being perceived as gatekeepers and to redress the power imbalance in professional contacts with transgender individuals. This categorization of measures and possibly addressed reasons for experiences of discrimination may be debatable, but it seems clear that the measures address different and varying numbers of these reasons. For a start, it would therefore be advantageous to implement those measures that have the highest chance of improving the most common reasons for transgender individuals’ experiences of discrimination. However, the fact that the measures mentioned by our interviewees largely coincide with those that are already well known and published in the literature is not least an indication that the implementation of these measures still leaves a lot to be desired. Greater efforts are needed to ensure that transgender individuals do not experience discrimination, especially when in contact with trans-specialists.

### Limitations

The study presented here has some limitations. Firstly, we only conducted interviews with a relatively small group of trans-specialists. However, our 20 interviews can be considered sufficient for qualitative interview studies. Typically, in qualitative interview studies, sufficient information can be obtained in just a few initial interviews, which result in data saturation [77, 78]. The 20 interviews we conducted therefore represented a sufficiently large number to answer our research questions. A further limitation results from the predominance of interviewees self-identifying as female and the relative preponderance of interviewees from psychiatry and psychotherapy. However, these proportions reflect the reality of the contacts that transgender individuals have with trans-specialists. Due to statistical realities, transgender individuals in Germany encounter practitioners self-identifying as female more frequently. As psychiatrists and psychotherapists play a significant role in the transition process of transgender individuals, they certainly have the most contact with them. In our opinion, the interviews we analyzed therefore reflect the reality of care for transgender individuals in Germany. There are indications that health professionals self-identifying as female have a more accepting attitude towards transgender individuals than those identifying as male [79]. Therefore, follow-up studies that explicitly investigate the influence of the gender identity of trans-specialists on their attitudes towards experiences of discrimination of trans individuals would be a useful addition. However, this was not the focus of the present study and must therefore be left to further research. Furthermore, it would also be interesting to analyze the response behavior depending on the professional group. Like the analysis of gender identity, this was not the focus of this study. Moreover, we would have had to work with more participants from the individual disciplines, particularly gynecologists and surgeons, in order to preserve the anonymity of the interviewees. The interviewees were all from Germany. A generalization of the results across the German cultural area is certainly not possible without further ado. Nevertheless, we assume that we were able to identify important aspects of discrimination against transgender individuals in contact with trans-specialists, which, although not fully transferable to other cultural areas, make an important contribution to research into discrimination against transgender individuals and should stimulate similar research projects in other countries. Finally, it could be considered a limitation that the interviews were only conducted by one researcher. However, this allowed us to conduct the interviews with the greatest possible standardization to achieve the most accurate and comparable results possible.

## Conclusions

To our knowledge, this is the first study that explicitly deals with experiences of discrimination of transgender individuals in contact with trans-specialists and that focuses on the perspective of trans-specialists. We were able to show various possible reasons why transgender individuals might experience discrimination specifically in contact with trans-specialists. First, gender identity, one of the core issues in contacts between transgender individuals and trans-specialists, is a very vague construct that has lost a great deal of definitional precision, particularly due to the identification of gender identities outside the male/female binary and various scientific uncertainties. These ambiguities or uncertainties regarding gender identity can lead to trans-specialist’s own, possibly implicit ideological assumptions or cultural stereotypes controlling behavior more strongly than desired. Hence, discussions about gender identity in therapeutic contacts with transgender individuals should be conducted with the utmost caution and in awareness of the conceptual vagueness of the term. Second, doubts on the part of trans-specialists as to whether or not their patients are transsexual contradict the attitude of most of our interviewees that the identification of the own gender identity lies solely in the autonomy of the individual itself. Such doubts could reactivate insecurities of transgender individuals and thus promote experiences of discrimination in contact with trans-specialists. Third, there is a lack of clarity regarding the classification of transsexuality as a mental illness. As a result, stigmatization in relation to mental illnesses can be mixed up with transsexuality and become effective. As the identification and diagnosis of transsexuality is an important part in the contact between transgender individuals and trans-specialists, this might be of specific significance regarding experienced discrimination on the side of transgender individuals. Fourth, our results imply that a trans-specialist’s own behavior, which may have had a discriminatory effect on transgender individuals, might be less remembered than it actually took place. As a result, trans-specialists may reflect on their own behavior less than necessary, which could lead to further discrimination experiences of transgender individuals. Hence, there is a need for trans-specialists to reflect more strongly on their own behavior with regard to potential discriminatory effects. This also includes becoming aware of different mechanisms through which discrimination experiences of transgender individuals could be caused. Additionally, in this study we have succeeded in compiling what we believe to be the most comprehensive list of causes currently being able to present that could lead to discrimination experiences of transgender individuals in contact with the healthcare system, in particular with trans-specialists. To reduce the possibility of experiences of discrimination in a transgender individual’s contact with trans-specialists, trans-specialists should be required to undergo self-experience sessions with regard to their own gender identity, be better oriented towards medical guidelines, regularly participate in intervisions and disclose roles and formulate mutual expectations at the beginning of therapeutic work with transgender individuals.

## Data Availability

The datasets generated and/or analyzed during the current study are not publicly available due to reasons of sensitivity, but are available from the corresponding author on reasonable request.
